# Modeling Flow and Mass Transfer within Hollow Fiber Packaging for Gas Separation

**DOI:** 10.1021/acs.iecr.5c03595

**Published:** 2026-06-22

**Authors:** Vimal Ramanuj, Ramanan Sankaran, Zamidi Ahmad, Fred Coan

**Affiliations:** † Computational Sciences and Engineering Division, 6146 Oak Ridge National Laboratory, 1 Bethel Valley Rd., Oak Ridge, Tennessee 37831, United States; ‡ Generon IGS, 922 Arcy Lane, Pittsburgh, California 94565, United States

## Abstract

Hollow fiber membrane modules are used for gas purification by their selective permeation properties. Intensification of the process to minimize the retentate loss and gas pressure involves optimization at various scales. In this work, we outline a numerical investigation of the gas separation performance at the scale of fiber bundles and its impact on module performance. Flow channeling and anisotropy govern the mass-transfer coefficient in axial and cross-flow configurations. These effects are quantified in terms of a permeability tensor or an anisotropy ratio and the effective mass-transfer coefficient or the Sherwood number. The results show a trade-off between purification and recovery. While smaller fibers offer a large specific surface area to enable high purification, it comes at a huge penalty on the separation performance due to reduced penetration within bundles. Optimum performance indicators are emphasized.

## Graphical abstract

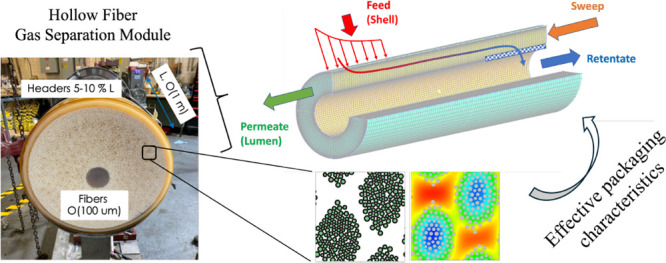

## 1 Introduction

Membrane gas separation technology finds application in oil and gas, chemicals, renewable energy, and decarbonization^1–3^ sectors among many others. Specific examples include production of He/N_2_ for inert gas consumers, ratio adjustment and H_2_ recovery from syngas (H_2_/CO) and biomass gasification streams, and methane purification in natural gas mixtures.^4,5^ The separation of CO_2_ from natural gas is of heightened interest due to the vast implications on manufacturing, energy, and economy. Natural gas constitutes a clean, efficient, and important fossil fuel source on the path to decarbonization of the energy sector and is the fastest growing primary source of energy in the global economy.^6^ Natural gas composition is highly dependent on its source. For example, natural gas obtained from oil fields can contain up to 90% CH_4_ with the remaining being mostly CO_2_,^7,8^ while that from reformed (gasified) biomass can have 35–65% CH_4_ along with significant CO_2_ mixed with sulfides.^9,10^ Its purification to meet distribution pipeline specifications becomes mandatory. Since CO_2_ is corrosive, reduces the heating value of natural gas, and can damage pumps when it freezes to dry ice, its composition is limited to less than 2 vol % in USA.^11^ Motivated by this challenge, we seek to develop a robust modeling approach capable of accounting for multiscale flow and mass-transfer features that have a compounding effect on the overall separation performance. Air separation (O_2_/N_2_ mixture) is well studied in the literature and forms a baseline for evaluating our approach.

Permeation flux through a porous membrane is driven by the difference in the chemical potential of constituent species across it. For an ideal gas, the flux is proportional to the partial pressure gradient. The proportionality constant consists of the permeance, which is a property of the membrane material. Selectivity is defined as the ratio of the large to small permeance or, in other words, the ratio of fast to slow species flux for a given partial pressure difference. This makes it energy efficient compared to thermally driven separation processes, such as distillation, that require a phase change. Separation modules typically consist of densely packed bundles of hollow fibers made of the membrane material. These have a unique advantage of providing a large specific surface area within a compact volume. At the same time, pressure drop over the length of a module and nonuniform flow distribution pose a challenge to its efficient operation.^12^ Membrane modules can be operated in three different configurations as shown in Figure 1: coflow, cross-flow, and counter-flow. These differ in the relative flow directions of the feed and permeate streams. Cross-flow patterns normally operate with feed streams requiring low pressurization but exhibit less-than-ideal efficiency. Counter-current configuration, in theory, is the most efficient as it provides a high uniform potential difference for mass transfer across the module length. Many gas applications require high shell-side pressurization for membrane durability reasons. A shell-side pressurized counter-current module can lead to stagnant flow patterns around the feed and product ends and incur too much pressure drop in forcing the counter currency. Improved computational models are required to predict the performance of gas separation modules for process and design optimizations and to aid the development of fabrication techniques that can balance the manufacturing complexity with process efficiency.^12,13^

**Figure 1:**
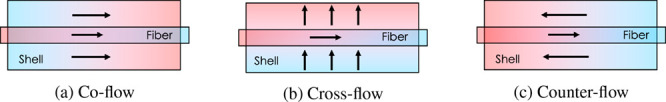
Flow configurations in a gas separation module. (a) Coflow, (b) cross-flow, and (c) counter-flow. The color represents the concentration of the retentate (slow) gas. Note that a counter-flow configuration has an almost uniform concentration difference (driving potential) throughout the module length.

Theoretical mass-transfer models show that the counter-current configuration provides the highest purity retentate and minimal loss of feed to the permeate side and requires the least membrane area^14^ to achieve a given purity. However, real modules operate far below these levels due to multiple sources of nonideality.^14,15^ A primary source of nonideality in the shell-fed module is the complex flow pattern within the module that causes the retentate and permeate to be in cross-flow over a significant portion of the membrane, rather than a counter-current flow. Figure 2 shows a fibrous packed membrane module. Flow distributions around the geometric features of the module such as headers, baffles, etc. are known to contribute toward the overall performance of the module.^13^ Further, flow and mass-transfer characteristics at smaller scales, i.e., within and around the fibrous bundles, also affect its performance significantly. Current one-dimensional plug flow^16^ and zonal models^14,17–20^ are insufficient to predict and optimize the flow pattern within a module and are unable to help achieve the operational efficiency of a true counter-current flow. For example, a representative model given by^14^ *ṁ*_*i*_ = – α_*i*_ *N*_*h*_δ*p*_*i*_ represents the mass-transfer rate *ṁ*_*i*_ for a species *i* in terms of its selectivity α_*i*_ and the partial pressure difference on the feed and lumen sides δ*p*_*i*_. The proportionality constant, *N*_*h*_, notionally represents the product of membrane permeance and the effective surface area. Note that various sources of nonideality are lumped together in this constant, which needs extensive calibration. Similar approaches exist in related domains such as water filtration, hemodialysis, etc.,^21,22^ where the mass-transfer model is described mathematically in terms of corresponding transport parameters, namely, permeability or resistance. A common challenge to characterize this effective transport behavior in terms of the underlying geometric features of the porous material or packaging persists. Significant pressure variations are expected around major design features such as baffles, ports, distributors, etc. Even more significant is the role of flow distribution within and around the fiber bundles that can severely deteriorate the effective surface area.

**Figure 2:**
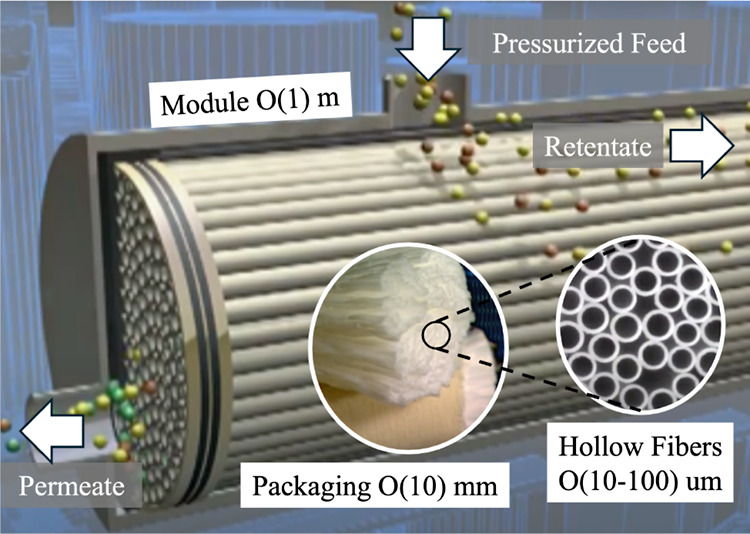
A typical hollow fiber module for gas separation application outlining the relevant features and length scales.

While computational fluid dynamics simulations can account for design features at a large scale, capturing the nonuniform flow distribution within the packaging remains a challenge. Recent literature^23,24^ has emphasized the role of nonuniform distribution around bundled packaging used in gas and liquid separations. The packaging was assumed to be homogeneous (random distribution) and impermeable (no flow penetration within bundle). A significant deviation from typical porous media models was observed even with these assumptions. Bundled packaging typically exhibits multiple discrete length scales over which flow features can be distributed. Variability in fiber diameter, spacing, and packing pattern are some of the geometric packaging features that can affect the flow distribution. Even small variabilities in fibers cause compounding effects in the flow and mass-transfer rates, thereby creating flow channels.^25^ At a larger scale, the effect translates into anisotropic flow resistance or permeability as has been demonstrated by the authors in similar purification applications.^26^ Current porous media empirical relations, based on nominal fiber diameters, are unable to predict the performance and retentate recovery in the presence of nonuniform flow and concentration fields.^14^ For example, Epstein^27^ showed that characterization of transport behavior in porous structures needs to account for tortuosity which itself is a flow-dependent quantity. Fiber-resolved direct numerical simulations (DNS) of the flow and mass transfer through bundles are necessary to analyze the effects of these nonidealities on mass-transfer rates. The works of Bao and Lipscomb^28,29^ and Sun et al.^24^ demonstrate the fiber-resolved mass-transfer models for random and ordered packing arrangements; however, real packing bundles have distinct characteristics that are not captured, namely, vastly different pore scales within and outside the bundles.

Motivated by the discrete length scales of existing numerical approaches and a typical homogenization approximation that is rarely applicable to anisotropic structures such as packed fibers, our goal is to bridge the gap through upscaling the knowledge gained from fiber-resolved simulations to module analyses. The two main objectives of this work are the following:

1. Quantify the anisotropic flow and mass-transfer properties for one specific realization of fiber bundle packing.
2. Evaluate its effect in representative module-scale CFD analyses.

The first part of this study focuses on deriving effective medium quantities such as packaging permeability and mass-transfer coefficient. Next, these are implemented in an air separation simulation where the goal is to verify its applicability to a large scale CFD. The numerical approach, formulation, and models are descried in the next section followed by the simulation results and analyses. Concluding remarks and opportunities for future work are also outlined.

## 2 Approach

The scope of our work includes mathematical formulation, performance analyses, and application of the model to an air separation test, although the method is generalizable for a much wider range of gas mixtures. The numerical modeling approach to capture the effects of fiber bundles on the flow and mass-transfer distributions and their impact on the module performance is outlined below. The method involves simulations at two distinct scales addressing the above-stated objectives: (1) fiber bundle scale resolving the fibrous packaging and (2) module scale where an effective porous media model is used. As a brief overview, the modeling strategy leverages fiber-resolved calculations and effective medium properties obtained from these analyses in the evaluation of model performance. The assumptions, geometries, physical model, and analysis quantities in each are described below.

Our focus lies in outlining a comprehensive modeling approach that highlights the role of multiscale characteristics. While distribution of packaging features such as fiber sizes, packing pattern, etc. are indeed significant, we limit the scope of this study to a geometry that captures the distinct pore scales, i.e., interfiber and interbundle sizes. Similarly, the module-scale simulation will also assume an elementary design such as a cylindrical shell and permeate channels. Inclusion of ports, baffles, distributors, etc. is common in engineering CFD analyses and is not considered here to keep simulations tractable. Predictive pore-scale and module analyses need to account for statistical variability in the geometric features, which can make computational problem sizes very large,^26^ outside the scope of this work. Note, however, that the following mathematical model is not restricted by such simplifications.

We begin by identifying the key length scales in the process as outlined in Figure 3. A typical gas separation module is shown in Figure 3a which is modeled as the annular region between two concentric cylinders. The outer and inner surfaces represent the shell or casing and the retentate channel, respectively. Permeate flows through the hollow fibers packed between these surfaces. Note that the module length is ∼1 m, and the cross section ∼10 cm. The fiber packaging is highlighted and a representative volume element (RVE) is shown in Figure 3b. It consists of several bundles that are ∼10 mm in size, while the fibers are ∼100 μm in diameter. The module and RVE geometries are used in the simulations.

**Figure 3:**
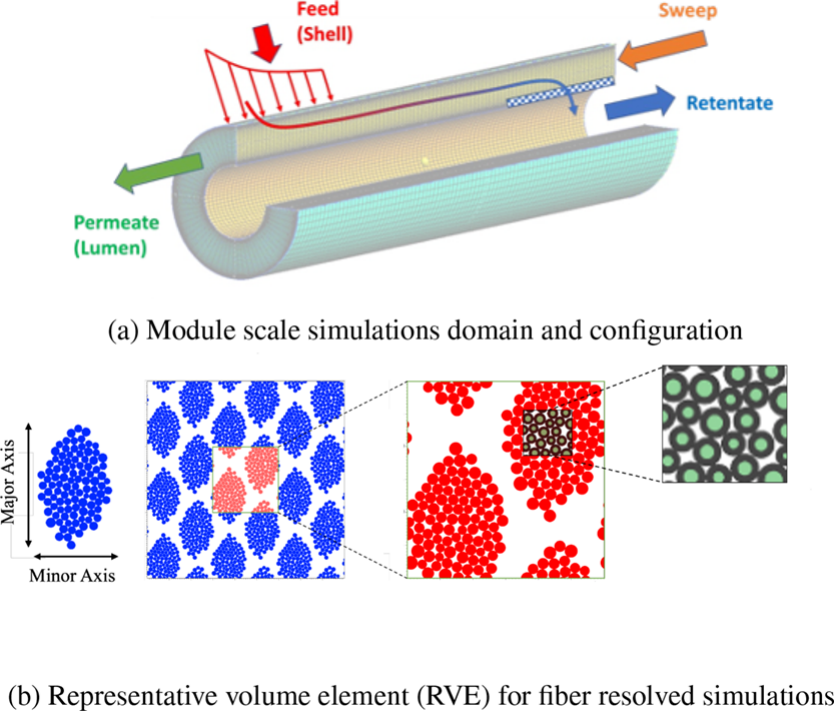
Problem setup and simulation domains. (a) Typical feed, permeate and retentate streams in a counter-current setup used in the module-scale simulations. (b) Porous structure formed by the fiber bundles. Various scales ranging from module (left) to individual fibers (right) are highlighted. The representative volume element (middle) resolves one periodic bundle which is used for the simulations.

A summary of the key physical quantities used in the mathematical modeling is presented in Table 1.

**Table 1:** Nomenclature Used in the Numerical Model^a^,^b^,^c^,^d^

symbol	quantity	comment
*X*, *Y*, *Z*	coordinates	*n* denotes wall normal coordinate
$\vec{U}$	velocity vector	
*R*	pore length scale	taken as approximate fiber size (100 μm)
*P*	pressure	
$\vec{G}$	applied pressure gradient	
*K*	permeability tensor	
*ṁ*	mass transfer rate	
*J*	molar flux	
*A*_f_	specific surface area	
*D*	siffusivity	
*Q*	permeance	
α	selectivity	fast to slow gas permeability ratio
ψ	mole fraction	
μ	viscosity	air viscosity for N_2_/O_2_ mixture
ρ	density	
*W*	molecular mass	
*C*	concentration field	
*q*_0_	molar flux	
*Re*	Reynolds number	uses *R* as reference length scale
*S*_*h*_	Sherwood number	denotes effective mass-transfer coefficient

a Subscripts in, out, feed, and w (wall): locations/conditions.

b Subscripts *i*, *j*, *A*, and *B*: species or indices.

c Superscript ^l^: lumen (permeate) side.

d Corresponding lower case or *^*denotes nondimensional quantity.

### 2.1 Fiber Bundle-Scale Simulations

The physical model and governing equations used for the fiber bundle scale simulations are described here. The RVE geometry outlined in Figure 3b is used. The geometry shows a periodic control volume that resolves two identical bundles with a random arrangement of hollow fibers. Each bundle consists of 50 fibers. The mean fiber diameter is ∼145 μm, and the packing factor is 0.45. The geometry will be referred to as the reference case, while other geometries with lower packing factors are also considered. The fiber surfaces are mathematically captured using a level set function distribution on a structured finite difference grid. Level set is basically a signed distance function with its magnitude representing the normal distance from the nearing fiber surface. The distance property is used to represent the physics (flow and mass-transfer boundary conditions) along the fiber surface using an embedded boundary formulation.^25^

Key assumptions are outlined below:

- The flow is well-developed so that entry effects on the velocity field are not considered.
- Cross-flow configurations are considered only for evaluating the flow distribution effects. Mass-transfer simulations assume an axial flow.
- The flow is decoupled from mass transfer. Mass-transfer rates are small enough that the flow distribution is treated as uniform in the axial direction.
- Axial diffusion is negligible compared with convection.

The flow, pressure, and concentration fields are represented in dimensionless form using the characteristic quantities proposed in ref 29. These are summarized below (also see Table 1).

$$\begin{array}{c} \begin{array}{ccc} x=\frac{X}{R}; & y=\frac{Y}{R}; & z=\frac{ZD}{\left(R^{4}/\mu )|\vec{G}\right|}\\ & \vec{u}=\frac{\vec{U}}{\left(R^{2}/\mu )|\vec{G}\right|}; & p=\frac{P}{|\vec{G}|R} \end{array}\\ c=\{\begin{array}{cc} \frac{C-C_{w}}{C_{0}-C_{w}} & \mathrm{const}.\;\mathrm{wall}\;\mathrm{concentration}\\ \frac{C-C_{0}}{q_{0}R/D} & \mathrm{const}.\;\mathrm{wall}\;\mathrm{flux} \end{array} \end{array}$$ 1

The dimensionless governing equations for the flow and concentration fields are given by eq 2.

$$\begin{array}{c} \vec{g}+\hat{\nabla }p={\hat{\nabla }}^{2}\vec{u};\;\hat{\nabla }\cdot \vec{u}=0\\ \vec{u}\cdot \hat{\nabla }c-{\hat{\nabla }}^{2}c=0 \end{array}$$ 2

The pressure gradient in this equation is decomposed into two components: $\vec{g}$ which is an average value for the RVE that is responsible for the net flow and ∇*p* which captures the effect of pressure distribution within the RVE domain due to the geometry. As has been mentioned earlier, direction of $\vec{g}$ is varied to represent axial and cross-flow configurations. It is worth noting that in a strictly axial flow configuration, the spatial pressure variation term is dropped, and the momentum equation reduces to that of refs 28,29 . In this work, cross-flow simulations are also performed to investigate anisotropic flow property where it becomes significant. A zero velocity condition is imposed on the fiber surfaces which is implemented using the level set model as described in ref 25.

Enrichment of the gas stream due to selective permeation can lead to lower mass-transfer rates downstream. This is a complex phenomenon and also depends on the distribution of the feed stream around the fibers. In this work, we consider two limiting conditions as also proposed by Bao and Lipscomb^28,29^ to compute the bounds of the effective mass-transfer coefficient.

- Constant wall composition: For fast sweep flow rates, the gas dilution/enrichment effects are negligible. In this case, the wall concentration is treated as constant *C*_0_. Mass-transfer characteristics are thus governed by the concentration gradient near the wall.
- Constant wall flux: A constant wall flux boundary condition given by *q*_0_ is representative of very small flow rates where wall dilution/enrichment progresses at the same rate as the feed flow. Nonuniform wall concentration limits the mass-transfer behavior.

With these assumptions, the boundary conditions along the fiber surface are given by eq 3.

$$\begin{array}{ll} \vec{u}=0; & dp/dn=0\\ c=0 & \mathrm{const}.\mathrm{wall}\;\mathrm{concentration}\\ dc/dn=1 & \mathrm{const}.\mathrm{wall}\;\mathrm{flux} \end{array}$$ 3

Domain boundary conditions are strictly periodic (including geometry).

### 2.2 Module-Scale Simulations

We leverage known CFD tools to implement the knowledge gained from fiber-resolved calculations at the module scale. Note that the following formulation is typical of a porous media flow solver with the exception of mass transfer and permeability representation. Module-scale simulations involve computing the feed/retentate and lumen side flow behavior characterized by the velocity, pressure, and fast gas mole fraction. Feed inflow conditions are given by the velocity *U*_feed_, pressure *P*_feed_, and the fast gas mole fraction ψ_*A*,feed_. Correspondingly, the Reynolds number is defined as *Re* = ρ*U*_feed_ *R*/μ. Lumen side is characterized by a constant pressure *P* ^*l*^.

The feed side momentum and continuity equations are given as

$$\begin{array}{ll} \rho (\vec{U}\cdot \nabla )\vec{U} & =-\nabla P+\nabla^{2}\vec{U}-K^{-1}\vec{U}+\dot{m}\vec{U}\\ \nabla \cdot \vec{U} & =\frac{\dot{m}}{\rho } \end{array}$$ 4

Assuming permeation flux driven by the partial pressure difference across the membrane, the mass-transfer rate, *ṁ* is given by eq 5.

$$\begin{array}{ll} \dot{m} & =A_{f}\Sigma W_{i}J_{i}\\ & =A_{f}\Sigma W_{i}Q_{i}(\psi_{i}^{l}P^{l}-\psi_{i}P) \end{array}$$ 5

*J*_*i*_ is the molar flux through the membrane material which is a product of the permeance *Q*_*i*_ and the partial pressure difference across the membrane for each species. For a binary gas mixture as the one considered here, selectivity α is defined as the ratio of fast to slow permeating species. Note the partial pressure difference becomes negative for the shell-side pressurized feed. The term *K* represents the permeability tensor of the fibrous porous medium. The molar conservation equation yields the following in terms of mole fraction ψ_A_. Note that we solve the transport equations for only the fast permeating species (*A*), and the mole fraction of (*B*) is given as 1 – ψ_A_(eq 6).

$$\rho \nabla \cdot (\vec{U}\psi_{A})=WA_{f}J_{A}$$ 6

Lumen side flow is assumed one-dimensional at a constant pressure. Thus, the flow velocity and composition are obtained by satisfying the continuity and molar conservation condition as

$$\begin{array}{ll} \frac{dU^{l}}{dZ} & =\frac{\dot{m}}{\rho^{l}}\\ \rho^{l}\frac{d(U^{l}\psi_{A}^{l})}{dZ} & =W^{l}A_{f}J_{A} \end{array}$$ 7

Note that *U* ^l^ is opposite to the feed stream, and thus, *ṁ* acts as a source term for lumen conservation equations. The module geometry is shown in Figure 3(a). The shell volume is treated as a uniform but anisotropic porous medium characterized by a permeability tensor. The feed inlet and outlet patches make up 10% of the module length with a fixed velocity inlet and a zero gradient pressure boundary condition applied, respectively. Note that the lumen flow is opposite to the net shell-side flow direction. A zero pressure gradient condition is imposed on the lumen outlet, and its opposite face is treated as an impermeable wall (zero normal velocity). Due to angular symmetry, only a finite sector of the module is considered with a cyclic boundary condition applied on the exposed faces (visible in Figure 3a). Eqs 4–7 are solved using the porous media solver in OpenFOAM.^30^ Mesh characteristics and sector sizes are determined using a convergence study discussed in Section 3.3.

### 2.3 Performance Evaluation

Flow and mass-transfer characteristics at the fiber bundle and module scale are sought. The key quantities of interest are defined below. As has been outlined in the introduction, the fiber-resolved simulations are performed to quantify the effect of bundle geometry on the flow distribution and mass transfer. Thus, the anisotropic permeability and specifically the anisotropy ratio are computed. Following the characteristic quantities defined in eq 1, the nondimensional permeability tensor can be represented as eq 8.

$${\hat{K}}_{ij}={\overset{-}{u}}_{i}|\vec{g}\cdot \hat{j}=1$$ 8

Recall, $\vec{g}$ is the externally applied unit pressure gradient. Thus, the permeability component *K̂*_*ij*_ is the mean flow observed in the *i* direction due to a pressure gradient applied in the *j* direction. For a strictly isotropic material, only the diagonal components are expected to be nonzero, i.e., the flow is observed only along the pressure gradient. However, fibrous bundles can introduce significant anisotropy due to the packing geometry. The flow anisotropy is quantified as the ratio of axial to cross-flow permeability and will be discussed further.

The mass-transfer characteristics are expressed in terms of Sherwood number *S*_*h*_ = *h*(2*R*)/*D* where *h* is the effective mass-transfer coefficient that accounts for sources of nonideality captured in fiber-scale simulations. Net mass transfer along the fiber surface satisfies^28^

$$S_{h}\int_{{\hat{A}}_{f}}(c-c_{w})da=\int_{{\hat{A}}_{f}}{\left(\frac{\partial c}{\partial n}\right)}_{w}da\Rightarrow S_{h}=\frac{\int_{A_{f}}(\partial c/\partial n{)}_{w}da}{\int_{A_{f}}(c-c_{w})da}$$ 9

Flux evaluated using the mass-transfer coefficient *h* and a more traditional expression using permeance *Q* yields

$$Q(P\psi -P^{l}\psi^{l})=h(C-C^{l})$$ 10

An illustration representing dimensional correlation is included in Appendix A.

The permeability ratio obtained from the above analyses is used in the module-scale simulations to solve for the feed/retentate and lumen side flow, pressure, and mole fractions. Using the results, key quantities of interest in describing the performance of the packing are the retentate recovery and purification and the separation performance. These are investigated for various membrane surface area and feed flow rates. For the slow permeating species (*B*), these are defined as eqs 11 and 12.

$$\mathrm{Recovery}=\frac{U_{\mathrm{out}}\psi_{B,\mathrm{out}}}{U_{\mathrm{in}}\psi_{B,\mathrm{in}}}$$ 11

and

$$\mathrm{Purification}=\frac{\psi_{B,\mathrm{out}}-\psi_{B,\mathrm{in}}}{1-\psi_{B,\mathrm{in}}}$$ 12

### 2.4 Code Description

The fiber-resolved flow and mass-transfer simulations are performed using the Quilt code.^25^ It uses a level set method to represent the fiber surface implicitly. The code uses a uniform finite difference method to solve the conservation equations. The fiber surfaces are considered fixed walls, and the corresponding embedded boundary conditions are implemented following a ghost fluid scheme outlined in ref 25. The same scheme is also used to quantify the mass-transfer coefficient that depends on the surface normal and surface concentrations as observed in eq 9. The RVE is periodic in lateral directions. The flow and composition fields are solved by using an operator split strategy. The pressure and velocity fields are first solved using a two-step projection method. Subsequently, the species transport is solved using an underrelaxed Gauss–Seidel operator. References 25,31 outline the grid dependence studies for similar applications. Following the results, the fibers are discretized with eight grid points across the diameter to capture the local surface normal flow and composition gradients. The code was executed in parallel on the CADES cluster at ORNL.

The module-scale analyses are performed using an OpenFOAM-based porous media solver. The solver is developed on top of *poroussimpleFOAM*.^32^ It uses a SIMPLE scheme to solve the shell-side momentum and continuity equations. The equations are solved implicitly using a Gauss–Seidel smoother and linearized source terms appearing in the momentum and mass conservation. The Laplacian and divergence operators are discretized using a second-order scheme with upwind stabilization. A convergence study is performed to investigate the effect of mesh size on the module performance results and determine an optimum resolution for the simulation cases. The mesh size is refined near the wall to resolve the high local flow gradients.

## 3 Results and Discussion

Simulation results discussed in this section are categorized into (1) fiber-bundle scale and (2) module scale. First, the setup outlined in Section 2.1 is used to perform flow simulations with and without mass transfer under cross- and axial-flow configurations. Major characteristics of interest in this study are the nonuniform flow and mass transfer across the fiber packing. Consequently, the effective medium properties, namely, the anisotropic permeability ratio and the mass-transfer coefficient, are evaluated. Further, the knowledge gained from these analyses is leveraged in module-scale simulations in Section 2.2 to analyze the effects on the flow distribution and pressure drop in a counter-flow configuration. Application to evaluate the gas separation performance for a representative mixture is reported. Specific geometric and operational parameters used in each case are outlined in the respective sections.

### 3.1 Flow Characteristics within Packaging

The fiber-resolved direct numerical simulations of flow (without mass transfer) are performed. Along with visualizations of the flow distribution, a prime objective is to quantify the effective medium behavior, given in terms of the permeability tensor. It is hypothesized that flow channeling arising due to the distinct length scales (see Figure 3) involved within a packaging, namely, interbundle spacing and within the bundle, leads to highly anisotropic permeability. Flow simulations are performed under axial and cross-flow configurations by varying the direction of the applied pressure gradient. Note that due to the elliptic bundle geometry, cross-flow cases consider pressure gradients in both major and minor axes.

Figure 4 shows the flow distribution and pressure contours in each of the cross-flow configurations. Visualizations demonstrate the flow channeling where a high velocity magnitude is observed in the interbundle spacing. Because of small pore sizes within the bundles, flow infiltration into it becomes more difficult. The effect is a manifestation of the two distinct pore length scales described above. The normalized permeability in these cases is 0.19 and 0.28.

**Figure 4:**
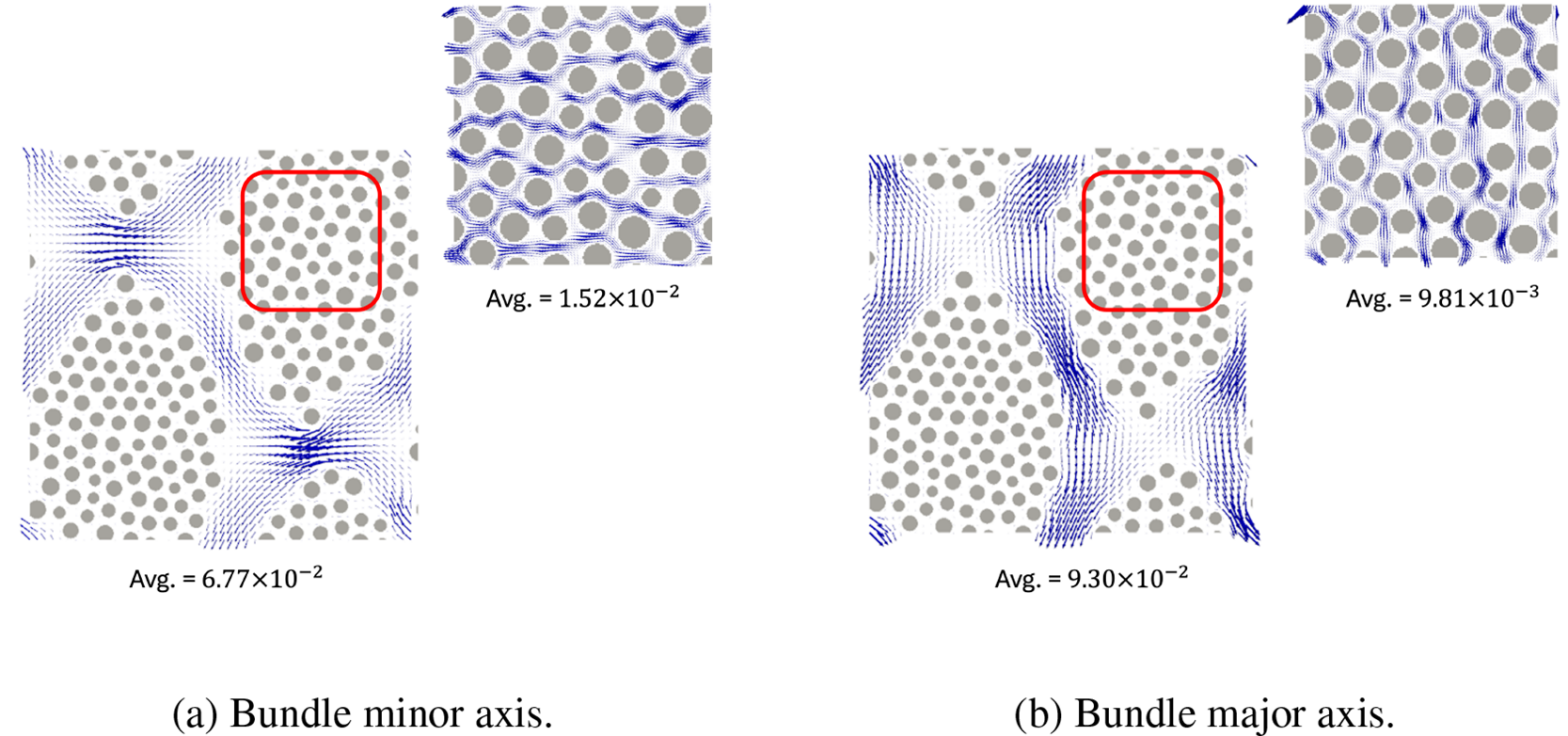
Flow channeling effects in cross-flow configurations. Two cases with pressure gradient applied along the (a) bundle minor and (b) bundle major axes are shown. Velocity vectors are shown along with average magnitudes in the RVE and within bundles (boxed region). Flow penetration within bundles is significantly less compared to interbundle channels.

Next, we consider an axial flow case where the pressure gradient and net flow is along the fiber axis. Along with the distribution of axial velocity component, the cross-flow is also visualized in Figure 5. Again, a high axial velocity is observed in the interbundle spaces compared to within a bundle. While the cross-flow velocity components are much smaller in magnitude, these also show similar distribution and is analogous to the cross-flow cases outlined earlier. The average flow in each direction is computed to evaluate the corresponding permeability components. The normalized axial permeability is 2.3 which is almost 12× and 8× greater than the cross-flow permeability observed in the previous cases (Figure 4).

**Figure 5:**
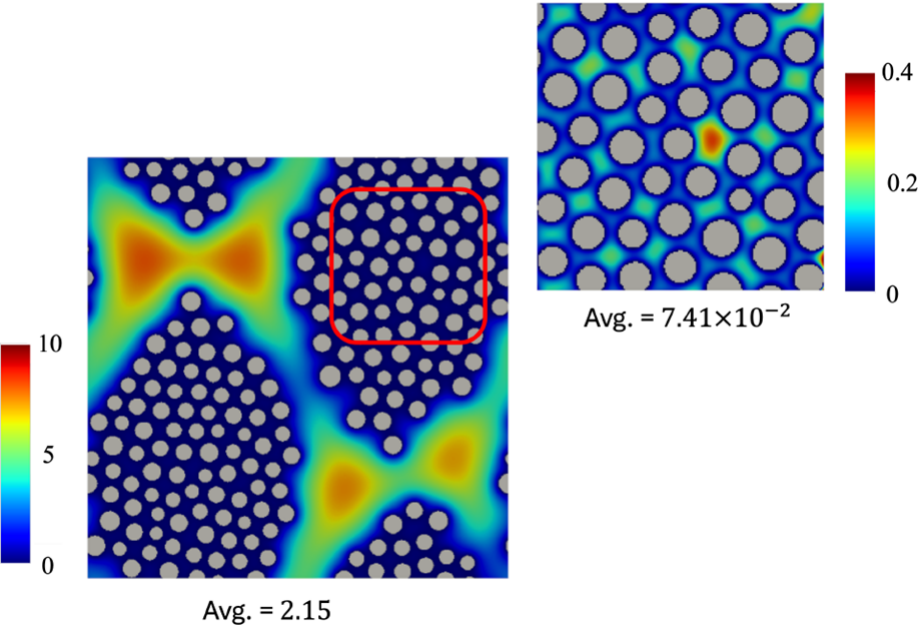
Flow channeling effect in axial flow configuration. Color represents the magnitudes of velocity components. Axial flux is significantly high within interbundle channels. Reduced flow penetration within bundles is realized in this configuration which impacts the overall mass-transfer characteristics.

Table 2 summarizes the flow characteristics. The key observation is that the anisotropy ratio varies between 12× and 8× depending on the cross-flow direction. These results emphasize the importance of capturing the relevant packaging configurations and length scales in modeling the flow behavior at a macroscale. It is also important to note that the nonuniform flow distribution within a packaging can have detrimental effect on the mass transfer and the overall module performance due to suboptimal infiltration into the bundle. The effect can be realized as under-utilization of the geometric surface area available for mass transfer. Further investigation of the impact of flow distribution and effective surface area on the module performance is presented later. Naturally, a simplified model in terms of the geometric properties of the packaging is insufficient to capture this characteristic behavior on a larger scale.

**Table 2:** Permeability Tensor from Fiber-Resolved Simulations Along with Corresponding Anisotropy Ratios^a^

axial	*K̅*_zz_ = 2.3	
cross	*K̅*_xx_ = 0.19	*K̅*_yx_ = 0.02
	*K̅*_*yy*_ = 0.28	*K̅*_*xy*_ = 0.02
anisotropy ratio	*K̅*_*zz*_/*K̅*_*xx*_ = 12	*K̅*_*xx*_/*K̅*_*yx*_ = 9.5
	*K̅*_*zz*_/*K̅*_*yy*_ = 8	*K̅*_*yy*_/*K̅*_*xy*_ = 14.0

a *x*: minor, *y*: major, *z*: axial (Figure 3b).

### 3.2 Mass-Transfer Characteristics within Packaging

In this section, we focus on the mass-transfer characteristics in an axial flow (flow field shown in Figure 5) that is relevant for typical counter-current arrangements. The axial flow solution from previous cases is used to solve for the concentration distribution for the two limiting cases described in Section 2.1. Results for the constant wall concentration and flux boundary conditions are presented in Figure 6. The primary objective is to analyze the effects of flow channeling on the mass-transfer coefficient.

**Figure 6:**
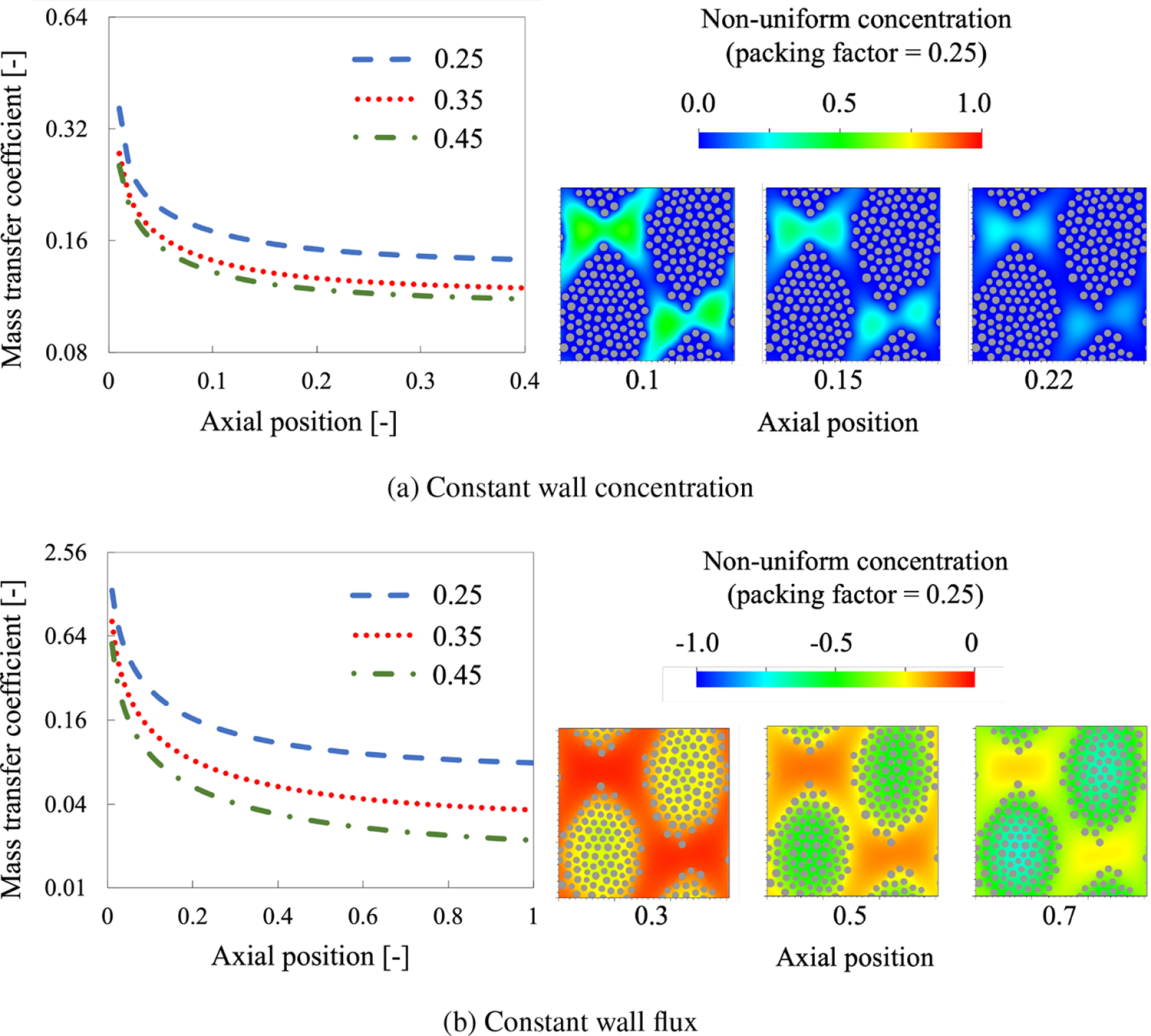
Mass-transfer characteristics at the fiber bundle scale in an axial flow configuration for constant (a) wall concentration and (b) wall flux conditions. The effective mass-transfer coefficient (Sherwood number) as a function of the axial position is shown along with a visualization of the concentration (see eq 1). Concentration distribution follows the flow channeling captured in Figure 5.

Mass transfer through the fiber walls is dependent on the competing effects of advection along the surface and permeation into the wall. The nonuniform axial flow distribution governs the local advection rate, while the permeation is dictated by the composition boundary condition. In either case, it is understood that the advection within the fiber bundle is extremely slow compared to that within the interbundle spacing. Consequently, a locally high concentration and reduced mass transfer are observed.

The mass-transfer coefficients expressed in terms of the Sherwood number are shown in Figure 6. The packing factor is varied between 25 and 45% by changing the fiber sizes (keeping the locations fixed). The results are shown along the length of the module *z*. As expected, *S*_h_ near the inlet is much higher (ignoring the entry effects) and converges to a lower value downstream. The converged values of the Sherwood numbers are given in Figure 7 for both limiting conditions. It is notable that the assumed conditions form the bounds of the mass-transfer characteristics. Assuming that a constant wall concentration represents a negligible dilution effect, flux is limited by the concentration gradient on the feed side and thus forms the upper limit. On the other hand, a constant wall flux condition represents a regime governed by dilution at the wall (ignoring entry effects), forming a lower bound for *S*_h_. Practical operation is expected to be bound by these limits and characterized by the feed flow rates. It can be seen that these values reduce monotonically with an increase in the packing fraction. The findings of Bao and Lipscomb^28,29^ using random and ordered packing show a distinct maximum in the corresponding trend. The packing used in their work involved a single characteristic length scale. In the case of fibrous bundles, the behavior is expected to be governed by channeling effects that diminish with an increase in the packing density.

**Figure 7:**
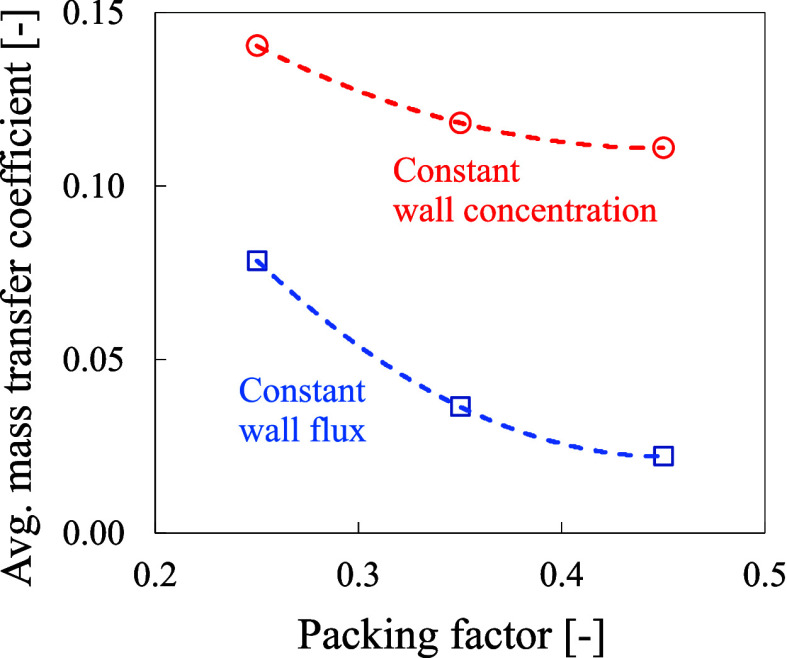
Average mass-transfer coefficient for various packing factors. The effective mass-transfer coefficient is high for sparse packing due to better (uniform) flow distribution within fiber bundles. The trend shows saturation beyond ∼40% packing density for a given configuration, indicating limited infiltration (flow channel in the interbundle space).

### 3.3 Gas Separation Performance

Having understood the importance of packaging effects on the effective flow and mass-transfer properties at the fiber bundle scale, we investigate how these translate into the performance of a larger scale module. A representative case with feed and operating parameters outlined in Table 3 is considered. The module-scale flow distribution in a counter-current arrangement is analyzed followed by the gas separation performance studies by varying the feed flow rate and the surface area effectiveness. Note that the permeance and anisotropy ratio are used based on observations in Sec. [Sec sec3.1] and 3.2 which inherit the flow and mass transfer nonuniformity observed at the packaging scale. An illustration of the correlation between the apparent permeance and mass-transfer coefficient observed from fiber-resolved calculations is included in Appendix A. First, we analyzed the numerical convergence of the results as functions of the discretized finite volumes and the resolved sector in the 3D geometry. Simulations resolving sectors with angles π/2, π, 3π/2, and 2π resulted in identical results; thus, a geometry with a quarter of the cross section was used for the analyses. Purification and recovery were analyzed for varying resolutions. Taking the finest resolution (20 × *R*) as a reference, Figure 8 presents second-order grid convergence in relative errors of each. Discretization corresponding to the 50 × *R* grid size was used for the following analyses.

**Figure 8:**
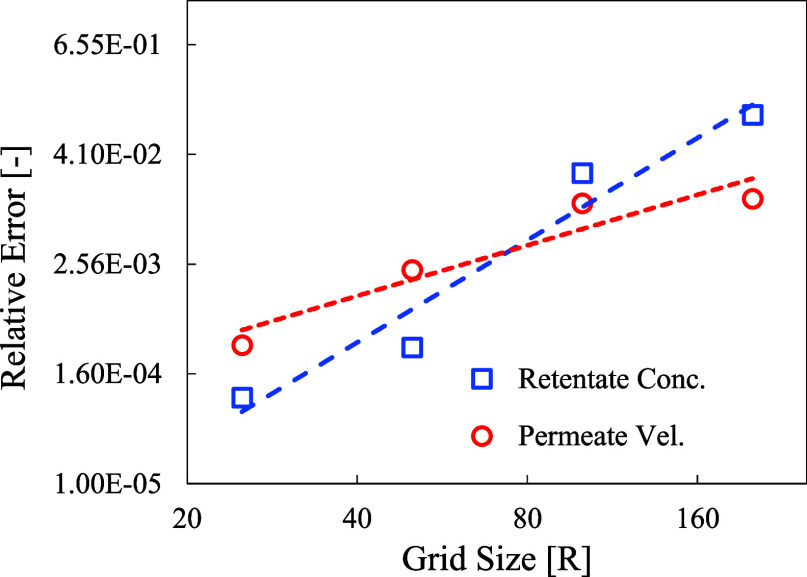
Second-order convergence in purification and recovery.

**Table 3:** Dimensionless Parameters Used for the Module Performance Evaluation^a^

parameter	value
Sherwood number^b^	0.05
selectivity	7
pressure ratio	16.67
feed Reynolds number^b^	1.25–40
feed mole fraction (fast gas)	0.21
normalized fiber surface area (*A*_f_/*A*_f,ref_)^c^	0.125–1
simulation results for ref case^c^	
flow rate ratio (feed to permeate)	0.33
permeate mole fraction (fast gas)	0.45
retentate mole fraction (fast gas)	0.07

a Flow permeability and packaging properties are used from fiber-resolved studies. Compositions correspond to fast permeating species. Simulation results are summarized for the reference case.

b See eq 10 and Appendix A example.

c Figure 3 and dimensions in Section 2.

Effects of anisotropy are incorporated by varying the ratio of axial to radial permeabilities. Figure 9 shows a qualitative visualization of the flow distribution by varying the radial and axial permeabilities compared to an isotropic case. As observed in the fiber-resolved simulations, axial permeability in realistic fiber bundles is expected to be O(10) greater than the radial component. Of major significance is the flow distribution near the outlet. A smaller radial permeability tends to distribute the flow more evenly across the outlet openings. A quantitative evaluation of this phenomenon can help in the performance assessments as discussed below.

**Figure 9:**
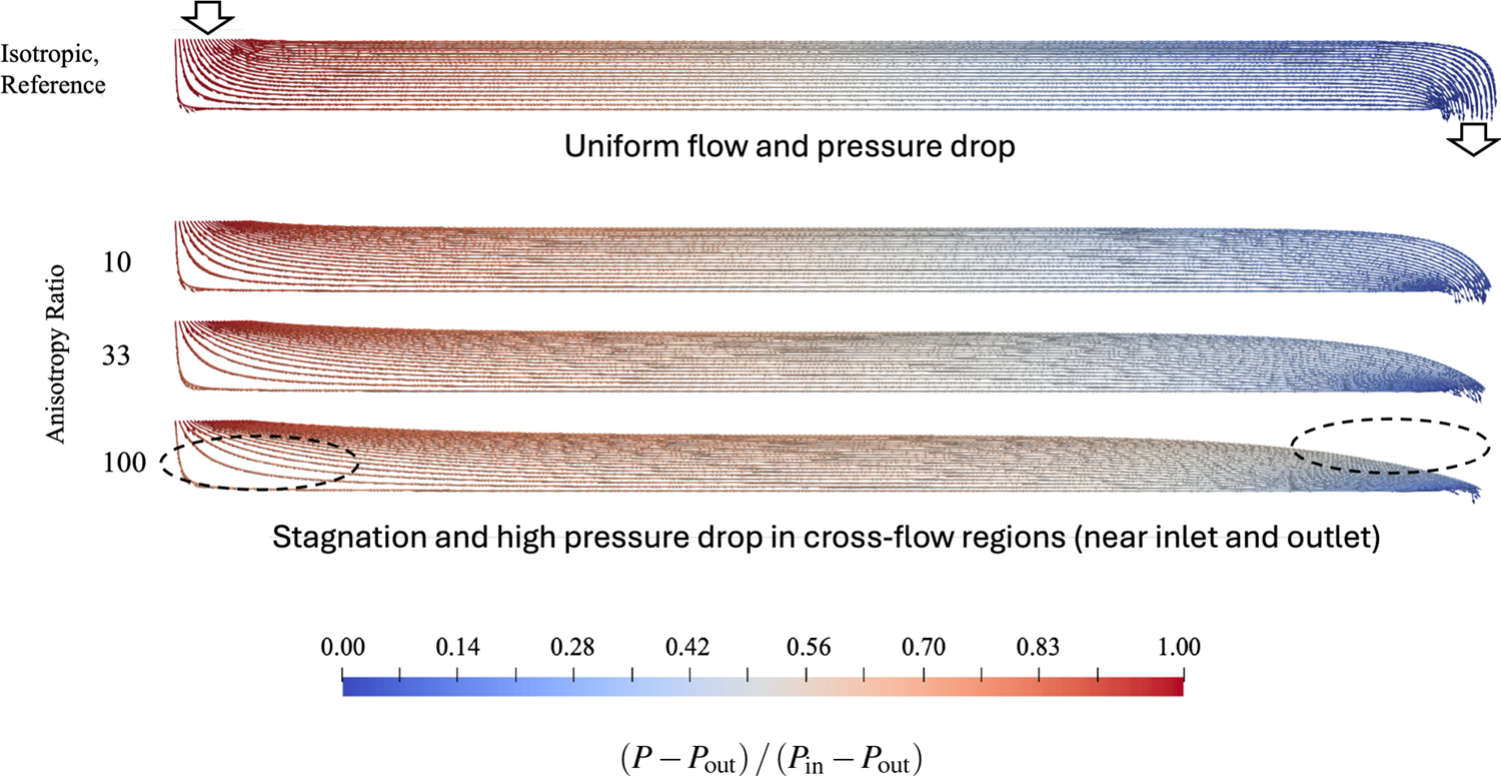
Effect of anisotropy ratio (up to 10×) on flow distribution. A 2D transverse slice of the module is shown (see Figure 3a for details). Streamlines are colored by the normalized velocity magnitude. The flow distribution indicates significantly under-utilized packaging volume near the ports for high anisotropy (i.e., small cross-flow permeability cases).

We vary the axial to radial permeability ratio by a factor of up to 100× to study the effects on flow distribution and especially near the outlet and the corresponding pressure loss. In Figure 9, the baseline isotropic case (high axial permeability) shows an even flow distribution across the entire length of the membrane module with an expectation of better utilization of the entire volume for mass transfer. However, the fiber-resolved simulations indicate that a realistic fiber packaging can have an anisotropy ratio of O(10). Correspondingly, we observe a detrimental effect on the uniformity of flow distribution. While the corresponding pressure drop also increases with anisotropy, the difference is only marginal. Pressure drop over a large module is primarily governed by the axial flow resistance.

Further, we include mass transfer in the model with a feed stream representative of an O_2_/N_2_ mixture and the membrane selectivity of 7 for O_2_. Reference parameters are taken from the nondimensional 1D model cases presented in refs 14,17 . First, the modeling results capture a trade-off between retentate recovery and purification as shown in prior works. Under plug flow assumption, i.e., radial homogeneity, ref 17 reports the convergence of purification to about 0.99 retentate mole fraction. In a 3D simulation that captures the nonuniform flow distribution, we observe a marginally lower mole fraction of 0.98 that corresponds to approximately 90% purification (see eq 12). Notably, that incurs a penalty on the retentate flow rate or recovery, which is too low to be practical.

We perform parametric simulations by varying the feed flow rate and the effective surface area, indirectly capturing the effects of reduced mass-transfer coefficient, as discussed in the fiber-resolved simulations in prior sections. The gas separation performance is examined in terms of the purification and retentate recovery in Figure 10a. The N_2_ recovery is compared against retentate purification for various specific surface areas. As the O_2_ permeates through the fibers, N_2_ enrichment occurs along the length of the module. Thus, further separation downstream becomes progressively difficult due to a lower partial pressure difference. Note that results from our model converge to approximately 0.98 N_2_ mole fraction, which is slightly lower than the 1D model predictions that overlook the nonidealities.^14,17^

**Figure 10:**
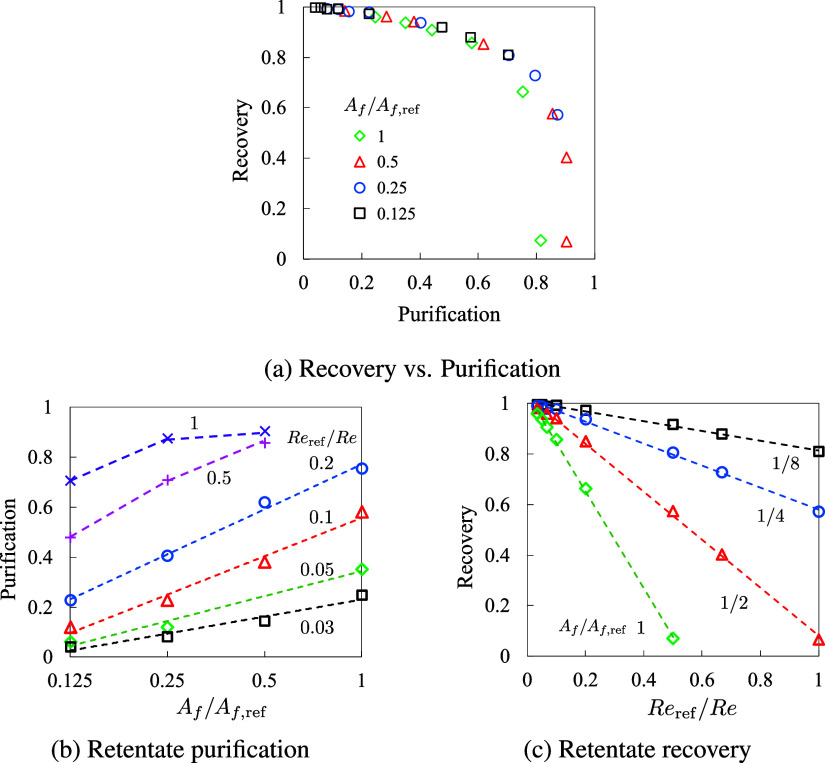
Module performance analyses for air (O_2_/N_2_) separation test. Results are presented in terms of retentate (slow gas, N_2_). (a) captures the recovery/purification trade-off behavior. Effects of fiber surface area and flow rates on (b) purification and (c) recovery.

Next, we perform a systematic study to investigate the influence of nonuniform flow distribution parametrized by the effective fiber surface area and the feed flow rate. The results in terms of purification and recovery are shown in Figure[Fig fig10]b,c. Looking at the reference surface area, we infer that purification reduces as the flow rate increases. Low flow rates offer longer residence times for the mass-transfer rate. However, beyond *Re*_ref_/*Re* ∼ 0.5, we see that the purification value almost saturates at its maximum which is 90% as discussed earlier. A decrease in the surface area has minimal effect. At high flow rates, purification varies almost linearly. For the entire range of surface areas considered, recovery is inversely proportional to the feed flow rate. Proportionality constant is a linear function of the surface area.

## 4 Conclusions

A multiscale modeling approach is applied to study the flow distribution and mass-transfer characteristics in a hollow fiber membrane module. Anisotropic flow distributions are hypothesized to be a major source of nonideality, significantly affecting the selective gas permeation performance in counter-flow configurations. Simulations consider a specific packing pattern that resolves multiple length scales, namely, interfiber and interbundle lengths. The resulting flow channeling effect highlights the role of these scales in governing the flow distribution by comparing with similar prior studies assuming homogeneous packing that overlooks the interbundle scale. The anisotropy ratio observed is approximately 8 – 12×, falling within the parametric range selected for the module-scale analyses. Next, the mass-transfer characteristics are studied under two limiting cases: constant wall concentration and constant wall flux. Theoretically, these conditions can be considered to form the bounds of mass-transfer behavior. The existence of distinct length scales appears to monotonically decrease the mass-transfer coefficient with increasing packing density, a behavior that is not captured by previous studies that do not resolve the multiple scales.

Next, we derive the effective medium permeability and the mass-transfer coefficient from the above and implement in module-scale CFD of an air separation test. It is noteworthy that we characterize permeation flux using the Sherwood number that accounts for the nonidealities at small scale. An illustration outlining its correlation with an apparent membrane permeance is also included. Results demonstrated severe flow pattern changes, especially near the shell outlet ports, as a function of varying anisotropic permeability ratio up to 10×. Particularly for high anisotropy, insufficient flow penetration can render a significant portion of the shell volume inefficient for mass transfer. Effects of feed flow rate and fiber surface area on the recovery and purification are highlighted. While the classical recovery-purification trade-off is observed, the reduced surface area can have a significant effect on the separation performance, especially at higher flow rates. A typical CFD study of a module can include complex design features which, integrated with our approach, becomes capable of accounting for locally cross-flow patterns through the permeability tensor and the nonideal permeate flux using the obtained mass-transfer coefficient.

## Appendix A

### Sherwood Number and Apparent Permeance

Here, we outlined an illustration demonstrating the dimensional correlation of apparent permeance *Q* and the mass-transfer coefficient characterized by the Sherwood number, *S*_*h*_. We assume the gas mixture to be O_2_/N_2_, and the physical properties are evaluated at feed conditions, i.e., 50 bar pressure and room temperature. Typical representative dimensions of a hollow fiber packaging are outlined in Table 4.

**Table 4:** Parameters and Physical Properties Used to Illustrate Correlation between Mass-Transfer Coefficient and Apparent Permeance^a^

quantity (Symbol)	value
Reynolds number (*Re*)	40
Sherwood number (*S*_h_)	0.05
porosity (ϕ)	0.5
pore size (*L*_pore_)	10^–4^ m
fiber size (*L*_fiber_)	2 × 10^–4^ m
module cross-section area (*A*)	0.03 m^2^
feed pressure (*P*_feed_)	5 × 10^6^ Pa
fast gas feed composition (*C*_feed_)	0.4 kmol· m^–3^
density (ρ)	60 kg· m^–3^
viscosity (μ)	2 × 10^–5^ Pa· s
diffusivity (*D*)	1 × 10^–7^ m· s^–1^

a An air separations test is used for example

The above *Re* corresponds to velocity, *U*, and flow rate, *F* = *U* × *A* × ϕ, obtained as

$$Re=\frac{\rho UL_{\mathrm{pore}}}{\mu }\Rightarrow U=0.13\;m\cdot s^{-1}\Rightarrow F=2\times 10^{-3}m^{3}\cdot s^{-1}=240\;\mathrm{sccm}$$

Next, the above *S*_*h*_ corresponds to

$$S_{h}=\frac{hL_{\mathrm{fiber}}}{D}\Rightarrow h=2.5\times 10^{-5}m\cdot s^{-1}$$

and using eq 10 at feed conditions (*C* ≫ *C* ^l^), apparent permeance is

$$hC_{\mathrm{feed}}=Q_{\mathrm{fast}}P_{\mathrm{feed}}\psi_{\mathrm{fast}}\Rightarrow Q_{\mathrm{fast}}=10^{-11}\;\mathrm{kmol}\cdot m^{-2}\cdot s^{-1}\cdot {\mathrm{Pa}}^{-1}=30\;\mathrm{GPU}$$
